# Supplementary material for: Comparative multiomics analysis of cell physiological state after culture in a basket bioreactor
Source: Sci Rep. 2022 Nov 23;12:20161. doi: 10.1038/s41598-022-24687-4 (PMC9686226; doi:10.1038/s41598-022-24687-4)
Supplement: Supplementary file 1 — Supplementary Information 1. [file 41598_2022_24687_MOESM1_ESM.zip › raw data/Proteomic raw data/6.KEGGenrichment/EG.vs.CG/ALL/pathway/src/04064.html]

map04064


- K03160

- Down regulated proteins

A0A0D9RQI3(Description:B-cell surface antigen CD40;Gene:CD40;log2FC:-1.3133)

- K11859

- Down regulated proteins

A0A0D9RWK4(Description:Ubiquitinyl hydrolase 1;Gene:TNFAIP3;log2FC:-1.0404)

- K06527

- Down regulated proteins

A0A0D9S6M6(Description:Uncharacterized protein;Gene:VCAM1;log2FC:-1.9990)

Close
